# Biological Matrices from *Cairina moschata* as Non-Destructive Biomonitoring Tools to Study Environmental Quality of Urban and Extra-Urban Areas: A Case Study of Palermo (Sicily, Italy)

**DOI:** 10.3390/ani13152474

**Published:** 2023-07-31

**Authors:** Matteo Riccardo Di Nicola, Christian Novello, Mario Lo Valvo, Gianluigi Maria Lo Dico, Vittoria Giulia Bianchi, Santo Raffaele Mercuri, Marcella Giornetti

**Affiliations:** 1 Unit of Dermatology and Cosmetology, IRCCS San Raffaele Hospital, Via Olgettina 60, 20132 Milan, Italy; dinicola.matteo@hsr.it (M.R.D.N.); bianchi.vittoriagiulia@hsr.it (V.G.B.); mercuri.santoraffaele@hsr.it (S.R.M.); 2 Asociación Herpetológica Española, Apartado de Correos 191, 28911 Leganés, Spain; 3 Dipartimento di Scienze Farmacologiche e Biomolecolari, University of Milan, Via Balzaretti 9, 20133 Milan, Italy; christian.novello96@gmail.com; 4 Dipartimento di Scienze e Tecnologie Biologiche, Chimiche e Farmaceutiche, University of Palermo, Via Archirafi, 18, 90123 Palermo, Italy; 5 Istituto Zooprofilattico Sperimentale della Sicilia “A. Mirri”, Via Gino Marinuzzi 3, 90129 Palermo, Italy; gigilodico@gmail.com

**Keywords:** anatidae, birds, biomonitoring, bioindicator, contamination, DMA-80, ICPMS, Muscovy duck, Mediterranean, allochthonous

## Abstract

**Simple Summary:**

Modern ecotoxicology analyses the biological material collected from animals to study the impact of the long-term exposure to contaminants on the environment. Among all contaminants, metals are particularly challenging to monitor, as they are typically present at trace levels. At the same time, toxicological concern is raised by their persistence in the organism and their possibility to be transferred through the food chain. Feathers could represent a non-invasive and valuable tool to study trace metal contamination, in view of their capability to accumulate trace elements over a long period of time. In the present study, feathers from the Muscovy duck were used to compare the levels of trace metals in two areas of Palermo (Sicily), one being in a central urban location and the other farther from the city centre. The comparison between feathers and blood samples also allowed to validate feathers as a suitable tool to monitor the long-term exposure to metals. Eventually, washing feathers with nitric acid could provide an insight on the actual concentration of metals accumulated within the feathers as a result of an intake as compared to the amount of metals deposited on them through air.

**Abstract:**

Biomonitoring is the qualitative observation and the measurement of biosphere parameters aimed at modelling the environment, evaluating its quality, and studying the effects of alterations on different ecological levels. In this work, trace metal concentrations were assessed using non-destructive biomonitoring tools as blood and feathers of the allochthonous aquatic bird *Cairina moschata*, collected within two areas of the Palermo metropolitan area, Sicily, differently exposed to air pollution: Parco D’Orleans, in a central urban location, and Monreale, southwest of the city centre. Higher concentrations in both blood and feathers collected in Parco D’ Orleans were found for lead, tin and selenium, but the same was not observed for other metals. The concentrations were not above physiological tolerance in any case. The comparison between blood and feathers allowed to realize that the latter are more useful for biomonitoring analyses, as they are indicative of both external contamination and bioaccumulation. Treatment with nitric acid highlighted that the feathers collected in Parco D’ Orleans had higher metal bioaccumulation than the ones collected in Monreale; however, the treatment needs standardization. The present study confirms that feathers and blood from *C. moschata* are a convenient and non-destructive sampling tool for metal contamination analysis.

## 1. Introduction

Biomonitoring is the qualitative observation and the measurement of biosphere parameters aimed at modelling the environment, estimating its quality, and studying the effects of an alteration on different ecological levels [1]. Modern ecotoxicology uses both wild and domestic animals as bioindicators to study the impact on whole ecosystems of the chronic exposure to multiple stressors interacting in a complex environment [2]. This new approach has the advantage to be more predictive of pollutants’ real bioavailability and of their consequences on natural and human systems, as compared to controlled-conditions experiments [3]. Nonetheless, the use of sentinel animal systems is subject to strict regulations in an effort to safeguard species and reduce animals’ distress [4]. For this reason, it is crucial to develop new non-destructive and non-invasive ways to evaluate biomarkers [5]. Over recent years, biomonitoring studies have widely focused on metals and metalloids [6,7]. In fact, among all the contaminants emerging from industrial activities, metals arouse particular toxicological concern, because of their capacity to bioaccumulate in tissues and biomagnify through the food chain [8,9].

The current study takes into account metals classified as heavy metals (Ag, Cd, Co, Mn, Hg, Ni, Pb, Cu, Sn, V, Zn) and heavy metalloids (As, Sb, Se), based on both density and toxicological assessments. These trace elements are mostly released from industries, urban settlements (i.e., wastewater discharges and sewage plants), mining, and agricultural activities [10]. They eventually pass to water basins, where they can undergo physical and chemical transformations, possibly enhancing their bioavailability [11]. Metals can be further subdivided into essential and non-essential, with the latter being more toxicologically relevant than the former ones. Indeed, not having a known physiological activity, non-essential metals are more prone to interfere with the metabolism and at the same time they are more difficult to excrete, potentially leading to various diseases and even cancer [12,13].

Previous literature is rich in trace metals biomonitoring studies using aquatic avifauna species [14,15,16]. Different reasons justify their convenience as bioindicators. First of all, birds have often demonstrated to be more sensitive to environmental contaminants than other vertebrates [17]. Additionally, water birds are preferred because of their diffusion in both natural and human systems [18], their high susceptibility and responsivity to metals [19], and the ease of sampling and handling them. Nonetheless, the majority of studies until now have been based on tissue samples collected during necroscopies [20,21,22].

The aim of the present paper is the validation of non-destructive biological matrices, collected from farmed birds, as suitable tools to analyse the difference in environmental quality of two areas of the Palermo province. The bird used is the Muscovy duck, *Cairina moschata* (Linnaeus, 1758), an Anatidae species of American origins. Blood and feathers were chosen as the samples to examine, because they are informative of two different exposure scenarios. Indeed, blood is a well-documented matrix for short-term metals biomonitoring, as it reports an exposure occurring in the two weeks prior to the analyses [23]. Vice versa, among all the non-destructive and easily accessible matrices, feathers are the ones presenting the best bioaccumulating capacity [24,25]. Concentrations measured in feathers may thus reflect a chronic exposure to environmental contaminants, even a prenatal one in the case of juvenile sampled individuals [26]. Additionally, they proved to positively correlate with the alterations of physiological functions [27]. It must be pointed out that both the type of feather and the area from which this is collected may affect the concentration assessment [28]. On this account, only the most studied feathers, i.e., the alar and the ventral ones, were investigated.

The final scope of research works such as the present one is to model ecosystems before they collapse, in ways as efficient as possible and compliant with the 3Rs principle. By all means, this has many implications for humans, according to the “one health” approach [29].

## 2. Materials and Methods

### 2.1. Sampling

**Figure 1:**
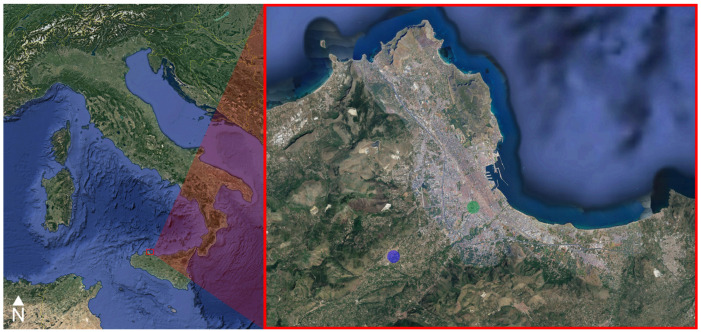
Map of Italy showing the location of the Palermo province (on the left). Focus on the urban area of Palermo, where Parco D’Orleans is located—green dot—and the peripherical area of Monreale—blue dot (on the right).

Samples were collected from 20 different *Cairina moschata* individuals of both sexes, half located in one of the aviaries at Parco D’Orleans and half owned by a private citizen in Monreale, from June to November 2018. The two sampling areas are representative of the city centre and the periphery of the Palermo province, respectively. Indeed, despite being rather close to the Palermo city centre (~4 km), Monreale has a separate municipality and is characterized by a much lower density of population (~73 inhabitants/km^2^ as compared to ~4000 inhabitants/km^2^), with consequently less car traffic and human activities, meaning that it could be used to model a moderately urbanized context (Figure 1).

The analyses were performed on 40 samples, consisting of:

- 10 blood samples drawn from the brachial vein in Parco D’Orleans;
- 10 blood samples drawn from the brachial vein in Monreale;
- 10 feather samples in Parco D’Orleans;
- 10 feather samples in Monreale.

Feathers were collected both from the alar region (primary, secondary, or covert) and from the ventral region.

Once collected, all samples were conserved in their sampling packaging and transported in refrigerated and labelled containers to the laboratories, where they were treated within one week after their arrival.

### 2.2. Sample Preparation

Refrigerated samples were brought to room temperature and prepared for extraction by homogenization. First, aliquots were collected from different points of the sample so as to obtain representative subsamples, which were then homogenized in a homogenizer with rotating porcelain blades. For mineralization, 1 g of sample was placed in a PTFE microwave vessel alongside 3 mL nitric acid ultrapur^®^ 60% (*v*/*v*) and 5 mL water. Digestion and complete oxidation of the organic material in the vessel were achieved through high-pressure focalized microwaves. The high-pressure microwave digester (model Multiwave 3000, Anton-Paar, Graz, Austria) allows for the complete oxidation of the organic material in the matrix by means of acid attack coupled with high-pressure focalized microwaves leading to strong oxidation. The instrumentation consists of a system for microwave emission (maximum power of 1400 W), a rotator for eight vessels, 100 mL hermetically sealed vessels in polytetrafluoroethylene, a software for effectively programming the digestions, a cavity for the rotor equipped with a suction system of the fumes, sensors for automatically monitoring temperature (which could possibly reach 300 °C) and pressure (reaching up to 1500 pounds per square inch), and vent valves. The software was configured for the programmed digestion to be suitable for various types of matrices and compliant with UNI EN 13805:2002 [30]. After vessel cooling, ultrapure water aliquots were used for sample recovery and for making up the volume to 50 mL. Ultrapure deionized water (resistivity = 18.2 MΩ cm) was obtained by means of Milli-Q^®^ Integral water purification system with Q-pod (Millipore, Bedford, MA, USA). After mineralization, metal concentrations were analysed following the procedure described in paragraph 2.3. The analysis was then repeated after washing the feathers with HNO_3_. More specifically, vessels were washed with a 5% nitric acid solution and then rinsed with Milli-Q water. Notably, both digestion and mineralization proved to be efficient without generating interferences with the following analyses. Particular attention was indeed paid in choosing metals and instrumentations free of active materials. For instance, each sample was filtered with a PTFE syringe filter (Ø = 0.45 µm). The residual samples were put again to conserve at T ≤ 20 °C.

### 2.3. Analytical Methods

Before performing the tests, the calibration curve was plotted according to the official method guidelines [31,32,33,34], i.e., by establishing at least 5 calibration points (blank included) and verifying the absence of interferences and of blank-imputable signals (R2 ≥ 0.99 for each analyte). Eventually, the concentration calculated by the software must not have more than a 10% deviation from the assigned theorical value.

Each sample was injected in duplicate and responded to the following condition:

|C_1_ − C_2_| ≤ r (1)

where C_1_ and C_2_ are the two concentrations and r is the limit of repeatability of the method at a level of concentration next to C_1_ and C_2_.

All the metals, except mercury, were analysed with ICP-MS (Inductively Coupled Plasma-Mass Spectrometry, PerkinElmer, Waltham, MA, USA), while mercury through the DMA80 (Direct Mercury Analyzer) technique, based on regulation (CE) n° 488/2014 [35] for cadmium level determination. Acceptability criterions are explicated in directive 2002/657/CE [36], UNI CEI EN ISO/IEC 17025/2005 [37], regulation (CE) n° 333/2007 [38] and regulation (CE) n° 836/2011 [39]. Quality parameters had to be compliant with the method UNI EN 15763:2010 [40]. The use of these two methods is well-documented for such analyses [41,42,43,44,45,46,47] and can be considered a gold standard.

Trace metals analysis with ICP-MS: The solutions for the ICP-MS analyses were prepared as summarized in Table 1. MRC solutions must have a purity ≥ 99% and the Internal Standard solution contains 6-Li, Sc, Ge, Rh, In, Tb, Lu, Bi. 100 mg/L in HNO_3_ 2%. The weighted sample (1 g) was withdrawn by an automatic sampler, combined with a quartz cyclonic nebulization chamber (cooled at 2 °C with water). The accuracy of the method was verified using the certified matrix DORM-4, produced by the National Research Council of Canada [48]. All the concentrations resulted within the acceptability interval, proving the validity of the method (Table 2).

Mercury analysis with DMA80: The solutions for the DMA80 analyses were prepared as summarized in Table 3. Initial sample weight = 0.1 g.

### 2.4. Data Analyses

The median, standard deviation, and maximum and minimum values of each element in all the samples were calculated with the STATISTICA 10 program. Verification of normality was performed with the Shapiro test. Given that not all samples showed a normal distribution, it was opted for the non-parametric Kruskall–Wallis test. Intra-laboratory repeatability and inter-laboratory reproducibility were calculated with the HORRAT_r_ and HORRAT_R_ formulas, respectively.

## 3. Results

### 3.1. Comparison between Sampling and Control Area

A total of 20 blood samples, 10 from Parco D’Orleans’ (urban area) specimens and 10 from Monreale’s (suburban area), were analysed for multi-trace metal quantification (Table 4).

**Figure 2:**
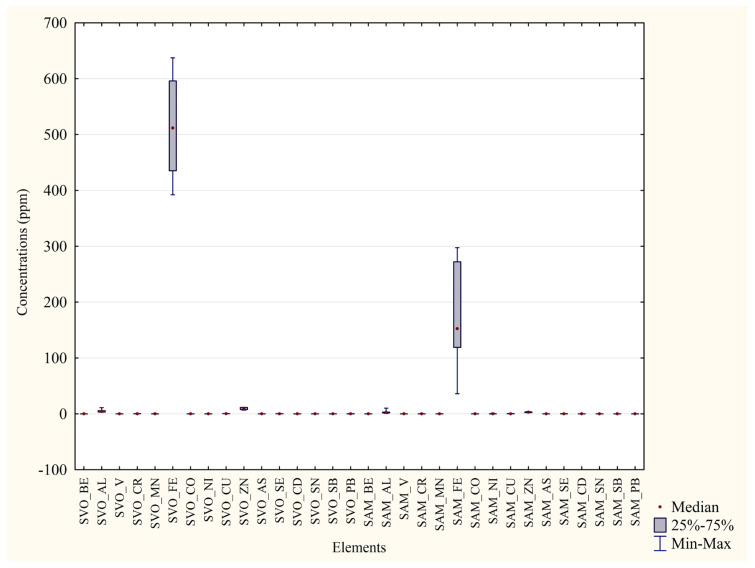
Comparison of blood concentrations in the two areas. Median, quartiles, minimum and maximum values of concentration (ppm) of each element in the two sampled areas. SVO = Blood from Parco D’Orleans specimens; SAM = Blood from Monreale specimens.

Results show higher concentrations in P. D’Orleans’ blood samples than Monreale’s ones for 10 elements out of 12, including all the non-essential toxic metals (As, Se, Sn, Pb). Nonetheless, only essential metals (Fe, Zn, Al) presented concentrations higher than 1 and a considerably high significance (*p* < 0.01 ** or *p* < 0.001 ***). In both matrices, mercury levels were too low to be compared with the other metals. The concentration difference between the two areas was particularly striking for iron, as represented in Figure 2. Correspondingly, 20 feather samples taken from the same individuals were examined (Table 5). It is noted that most metals’ concentrations were higher in the Monreale’s than in the P. D’Orleans’ samples, with only three non-essential metals such as Se, Sn, and Pb actually providing an opposite result. In the case of these latter elements, it is still relevant to observe that higher concentrations in the feathers (Table 5) as compared to the blood samples (Table 4) were found, attesting a possible bioaccumulation process.

**Figure 3:**
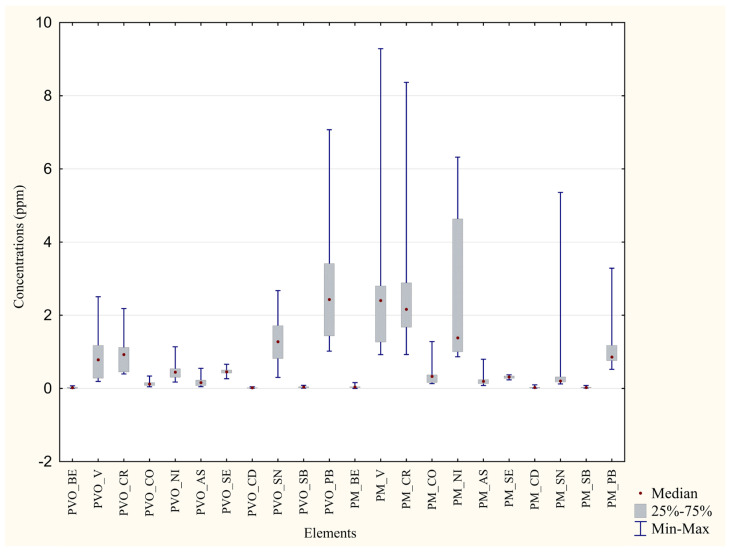
Comparison of feathers concentrations in the two areas. Boxplot representation of the metals concentrations (in ppm) found in the P. D’Orleans’ and the Monreale’s feathers. PVO = Feathers from Parco D’Orleans specimens; PM = Feathers from Monreale specimens.

It is also acknowledged that feather data presented a slightly lower statistical significance, a considerable variability (Figure 3), and only a few metals presented concentrations higher than 1. Mercury’s concentrations were not quantitatively relevant; thus, they are not shown.

### 3.2. Comparison between Different Matrices from the Same Individuals

**Figure 4:**
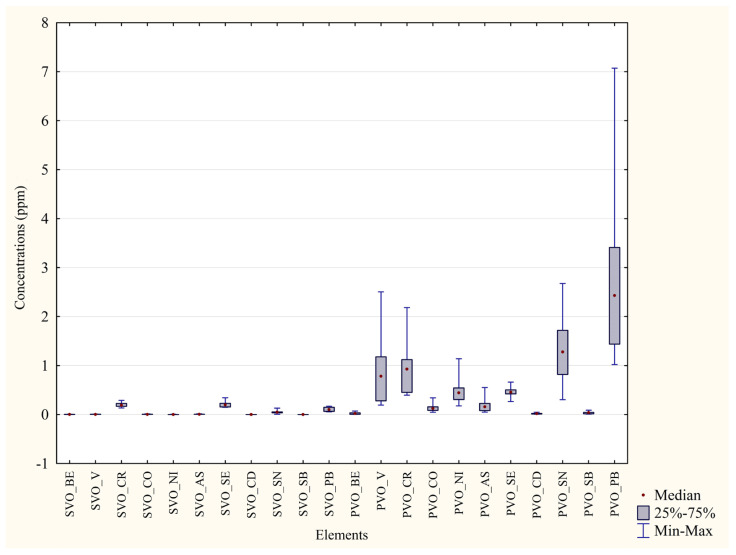
Comparison of concentrations in the two matrices from P. D’Orleans. ppm concentrations of trace metals in blood and feathers from P. D’Orleans. SVO = Blood from Parco D’Orleans specimens; PVO = Feathers from Parco D’Orleans specimens.

**Figure 5:**
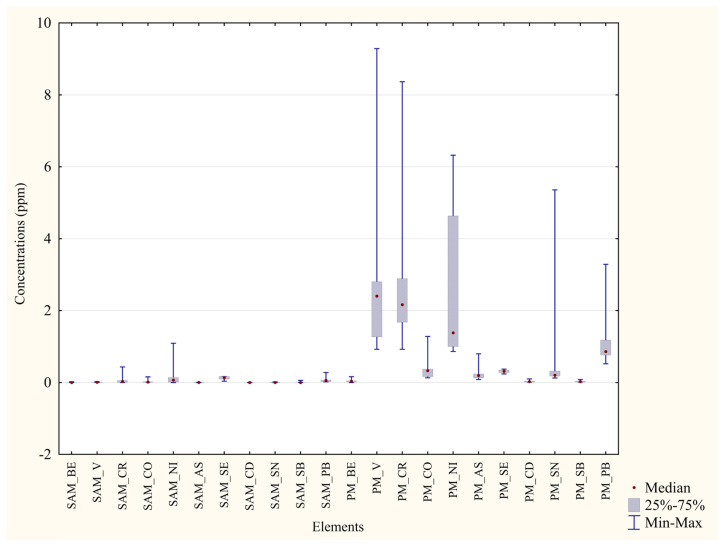
Comparison of concentrations in the two matrices from Monreale. ppm concentrations of trace metals in blood and feathers from Monreale. SAM = Blood from Monreale specimens; PM = Feathers from Monreale specimens.

Metal concentrations from blood and feathers of each area were compared with the aim of ascertaining which is the matrix with the best bioaccumulating capacity (Table 6 and Table 7). As depicted in Figure 4 and Figure 5, all the percentage differences in concentration were marked, even more than the differences measured with the same matrices of different areas, and *p* values were highly significant (***) for all the metals under analysis. Every median value was higher in the feathers than in blood.

### 3.3. Comparison between Washed and Unwashed Feathers

Washing with HNO_3_ was aimed at establishing whether the so-far-analysed concentrations must be entirely ascribed to the bioaccumulation process, or whether they partly result from the atmospheric superficial deposition of particles.

The metal concentrations in the feathers before and after washing them with HNO_3_ are set side by side in Table 8 and Table 9.

**Figure 6:**
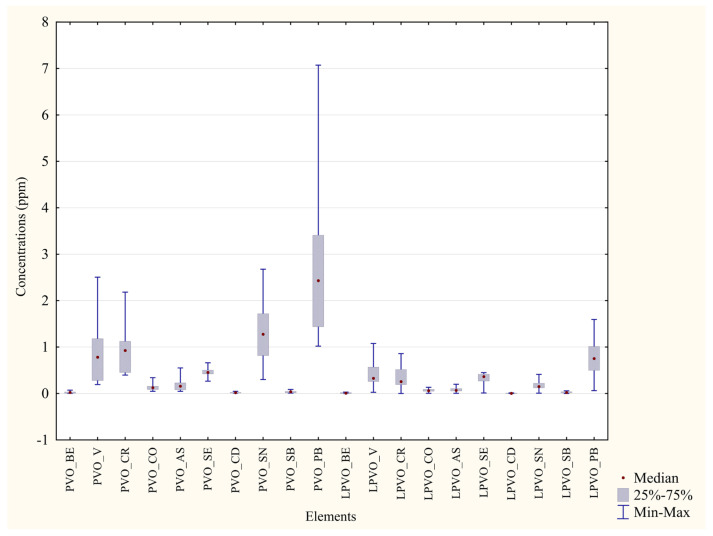
Comparison between the metal concentrations in unwashed and washed feathers collected in P. D’Orleans.

**Figure 7:**
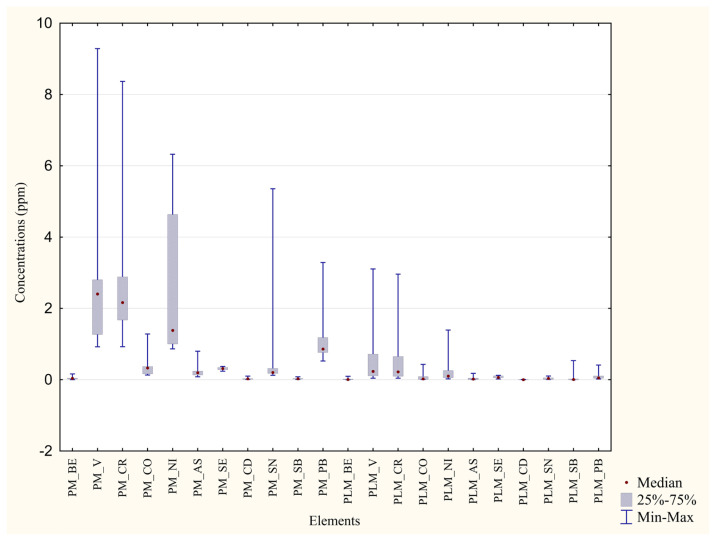
Comparison between the metal concentrations in unwashed and washed feathers collected in Monreale.

Percentage differences were high for both the sampled areas (always more than 80% for Monreale, for example) (Figure 6 and Figure 7) and *p* values obtained from the statistical tests were overall highly significant. It follows that the treatment with HNO_3_ was able to remove a substantial part of trace metals.

## 4. Discussion

### 4.1. Comparison between Sampling and Control Area

The results of the multi-trace elements analysis performed in parallel on the two areas show a statistical correlation between the sampled site and the level of contamination. Even though C. moschata demonstrated a great ecological flexibility, blood sample tests clearly indicate higher metal concentrations in P. D’Orleans, with significant *p* values. Statistically relevant difference in concentration, where present, may be explained by a possible contamination of the more urban location (i.e., P. D’Orleans), especially for those non-essential metals that are not subject to natural uptake through the diet or water. In fact, essential metal concentrations are influenced by periodical fluctuations due to pollution, as well as the diet and the periods of moulting [49,50]. As evidence of this, the only metals whose trend is concordant between the two matrices are some non-essential ones, such as Sn, Se, and Pb. It should be noted that no concentration exceeds the limit of safety. For example, lead, which is the most studied non-essential metal in biomonitoring, is present in all the individuals of both areas, but concentrations seem to be not of concern (provided that in ducks they should not exceed 0.39 ppm [51], whereas values oscillate between 0.054 and 0.17 ppm in P. D’Orleans and between 0.017 and 0.27 ppm in Monreale).

### 4.2. Comparison between Different Matrices from the Same Individuals

The second part of this study focused on the validation of feathers as a possible tool to assess bioaccumulation, as opposed to blood. From the comparison of the results obtained in the two matrices, it appears clear that feathers have a higher bioaccumulation capacity than blood. It can be inferred that feathers are more suitable for studying environmental quality in the long term. Indeed, the capacity to bioaccumulate low concentrations of metals over time makes feathers a better indicator of chronic exposure [14]. On the contrary, blood appears to be the optimal tool to evaluate acute exposures, due to the chelating potential of the metallothioneins contained in it [52].

### 4.3. Comparison between Washed and Unwashed Feathers

**Figure 8:**
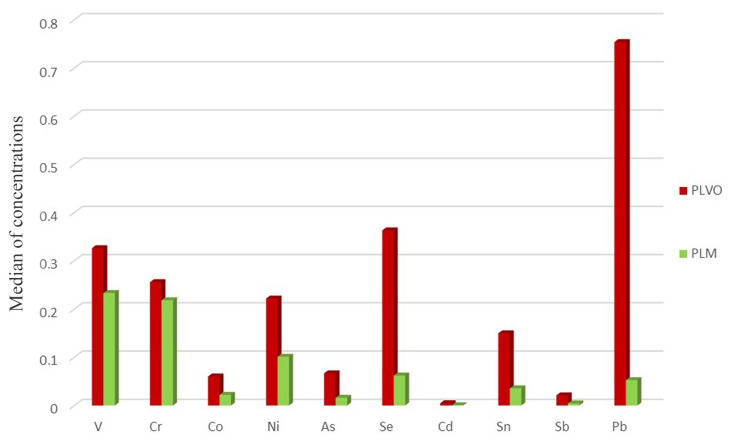
Metal concentrations after washing. Histogram illustrating the comparison of the metal concentrations (ppm) from Parco D’Orleans (PLVO) and Monreale’s (PLM) feathers after the treatment with HNO_3_.

The treatment with HNO_3_ and the differential analysis of the washed and unwashed feathers are relevant because they give the opportunity to capture the percentage of contamination deriving from the actual intake of metals (absorption, through an endogenous route) and the contribution of superficial deposition (adsorption, through an exogenous route). Indeed, data from unwashed feathers give a hint of the total exposure in each area, deriving from both atmospheric and tissue contamination, whereas washed feathers inform exclusively on the bioaccumulated concentration. The removal of the adsorbed fraction, even if it cannot be total with the current methods, led to a decrease in all the metal levels, especially those of some toxic metals such as Sn, Cd, and Pb (Figure 8). It is also worth mentioning that not all the metals react the same way to the treatment with HNO_3_ [53]. Notably, results collected after washing confirmed an overall higher presence of trace elements in the P. D’Orleans area, and this may be reconducted to its urban location. As a last note, it is acknowledged that several factors that are potentially able to influence the bioaccumulation capacity of feathers such as sex, age, metabolism, and period of moulting [49,50] were not taken into account in the present analysis. Concentrations may also vary depending on the type of feather and the section of feather taken as a sample. In addition, the potential difference between wild and farmed birds could be studied. Eventually, some authors agree on the possibility that the intake of adsorbed metals may be higher in water birds, due to the secretion of trace elements from their glands and the mechanical rubbing action of their beak [54]. All these factors potentially impacting the suitability of the use of feathers for biomonitoring purposes may be addressed in future works. The authors of this study indeed encourage future analyses where these elements are specifically studied, with the final aim to minimize variability and standardize the technique.

## 5. Conclusions

The current study suggests two non-destructive and easily accessible matrices, such as blood and feathers, as valid tools for short- and long-term biomonitoring assays, respectively. The use of feathers allows for an in-depth analysis of the contribution of the exogenous and the endogenous routes on the contamination with trace metals. Ultimately, the comparison between these two matrices is a valuable way to study contaminant fluctuations in those sites in which air quality monitoring stations are present. Even if the use of feathers needs standardization, the authors consider it worthy of further investigations and, in particular, they propose to use biological matrixes collected from non-aquatic avifauna.

## Figures and Tables

**Table 1:** Summary of the solutions prepared.

Solution	Concentration	Volume and Solution for Extraction		Final Volume (Flask)	Solvent
MRC	1000 mg/L				H_2_O/HNO_3_ 2%
MR1	10 mg/L	100 μL	MRC	10 mL	H_2_O/HNO_3_ 2%
MR2	1 mg/L	1000 μL	MR1	10 mL	H_2_O/HNO_3_ 2%
MR3	500 mg/L	500 μL	MR1	10 mL	H_2_O/HNO_3_ 2%
MR4	100 µg/L	1000 μL	MR2	10 mL	H_2_O/HNO_3_ 2%
MR5	50 µg/L	1000 μL	MR3	10 mL	H_2_O/HNO_3_ 2%
MR6	10 µg/L	1000 μL	MR4	10 mL	H_2_O/HNO_3_ 2%
MR7	5 µg/L	1000 μL	MR5	10 mL	H_2_O/HNO_3_ 2%
MR8	1 µg/L	1000 μL	MR6	10 mL	H_2_O/HNO_3_ 2%
MR9	0.5 µg/L	1000 μL	MR7	10 mL	H_2_O/HNO_3_ 2%
MR10	0.1 µg/L	1000 μL	MR8	10 mL	H_2_O/HNO_3_ 2%
MR11	0.05 µg/L	1000 μL	MR9	10 mL	H_2_O/HNO_3_ 2%

**Table 2:** Results from the certified matrix DORM-4.

Element	Measured Value (mg/kg)	Assigned Value (mg/kg)	Acceptability Interval (mg/kg)
Cadmium	0.291 ± 0.030	0.299	0.281–0.317
Lead	0.392 ± 0.040	0.404	0.342–0.466
Arsenic	6.980 ± 0.004	6.87	6.43–7.31

**Table 3:** Summary preparation of the mercury solutions.

Solution	Concentration	Volume and Solution for Extraction		Final Volume (Flask)	Solvent
MRC	1000 mg/L				HCl 5%
MR1	10 mg/L	100 μL	MRC	10 mL	HCl 5%
MR2	1 mg/L	1000 μL	MR1	10 mL	HCl 5%
MR3	100μg/L	1000 μL	MR2	10 mL	HCl 5%
QC2	20 μg/L	200μl	MR2	10 mL	HCl 5%
QC3	500 μg/L	500 μl	MR1	10 mL	HCl 5%

**Table 4:** Percentage differences between metal concentrations (ppm) from the two different blood samples. Only metals showing moderately significant (*p* < 0.05 *), significant (*p* < 0.01 **), or highly significant (*p* < 0.001 ***) results were reported.

Metal	Blood P. D’Orleans ±Median min–max (ppm)	Blood Monreale ±Median min–max (ppm)	Significance (*p* Value)	Percentage Difference between the Two Areas
Al	±4.27 2.92–11.15	±2.62 0.98–10.24	*p* = 0.0082 **	38%
Cr	±0.19 0.13–0.29	±0.031 0.00–0.43	*p* = 0.02 *	83%
Mn	±0.12 0.069–0.46	±0.061 0.016–0.28	*p* = 0.0156 *	48%
Fe	±511.81 392.08–637.37	±152.61 36.25–297.52	*p* = 0.0002 ***	70%
Co	±0.0035 0.0016–0.014	±0.012 0.0079–0.16	*p* = 0.0012 **	71%
Ni	±0.00 0.00–0.0069	±0.068 0.00–1.09	*p* = 0.0007 ***	100%
Cu	±0.33 0.28–0.51	±0.27 0.19–0.39	*p* = 0.004 *	19%
Zn	±9.27 6.78–11.66	±3.06 2.10–4.18	*p* = 0.0002 ***	67%
As	±0.0049 0.0038–0.014	± 0.0018 0.00–0.0044	*p* = 0.0012 **	62%
Se	±0.20 0.15–0.34	±0.13 0.038–0.17	*p* = 0.0041 **	33%
Sn	±0.048 0.0048–0.13	±0.00 0.00–0.018	*p* = 0.003 ***	100%
Pb	±0.10 0.055–0.17	±0.059 0.018–0.28	*p* = 0.0413 *	41%

**Table 5:** Percentage differences between metal concentrations (ppm) from the two different feather samples. Only metals showing moderately significant (*p* < 0.05 *), significant (*p* < 0.01 **), or highly significant (*p* < 0.001 ***) results were reported.

Metal	Feathers P. D’Orleans ±Median min–max (ppm)	Feathers Monreale ±Median min–max (ppm)	Significance (*p* Value)	Percentage Difference between the Two Areas
Al	±402.24 79.67–1472.25	±1260.76 550.71–5668.88	*p* = 0.0041 **	68%
Cr	±0.93 0.40–2.18	±2.16 0.93–8.37	*p* = 0.0052 **	57%
V	±0.78 0.19–2.51	±2.40 0.92–9.29	*p* = 0.0032 **	67%
Fe	±330.29 90.84–1237.44	±863.91 344.35–4217.01	*p* = 0.01 **	62%
Co	±0.12 0.048–0.34	±0.33 0.13–1.28	*p* = 0.0082 **	62%
Ni	±0.44 0.18–1.14	±1.38 0.87–6.32	*p* = 0.0004 ***	67%
Cu	±9.42 7.63–10.86	±7.44 5.75–12.77	*p* = 0.049 *	21%
Se	±0.46 0.27–0.66	±0.31 0.24–0.37	*p* = 0.0015 **	31%
Sn	±1.28 0.30–2.68	±0.20 0.13–5.36	*p* = 0.0052 **	84%
Pb	±2.43 1.02–7.07	±0.86 0.52–3.29	*p* = 0.0032 **	64%

**Table 6:** Comparison between the metal concentrations in blood and feathers collected from P. D’Orleans. Only metals showing highly significant (*p* < 0.001 ***) results were reported.

Metal	Blood P. D’Orleans ±Median min–max (ppm)	Feathers P. D’Orleans ±Median min–max (ppm)	Significance (*p* Value)	Percentage Difference between the Two Areas
V	±0.0042 0.0028–0.0081	±0.78 0.19–2.51	*p* = 0.0002 ***	99%
Cr	±0.19 0.13–0.29	±0.93 0.40–2.18	*p* = 0.0002 ***	79%
Co	±0.0035 0.0016–0.014	±0.12 0.048–0.34	*p* = 0.0002 ***	97%
Ni	±0.00 0.00–0.0069	±0.44 0.18–1.14	*p* = 0.0001 ***	100%
As	±0.0049 0.0038–0.014	±0.16 0.048–0.55	*p* = 0.0002 ***	96%
Se	±0.20 0.15–0.34	±0.46 0.27–0.66	*p* = 0.0002 ***	56%
Cd	±0.00043 0.00033–0.00076	±0.017 0.0081–0.047	*p* = 0.0002 ***	97%
Sn	±0.048 0.0048–0.13	±1.28 0.30–2.68	*p* = 0.0002 ***	96%
Pb	±0.10 0.055–0.17	±2.43 1.02–7.07	*p* = 0.0002 ***	95%

**Table 7:** Comparison between the metal concentrations in blood and feathers collected in Monreale. Only metals showing significant (*p* < 0.01 **), or highly significant (*p* < 0.001 ***) results were reported.

Metal	Blood Monreale ±Median min–max (ppm)	Feathers Monreale ±Median min-max (ppm)	Significance (*p* Value)	Percentage Difference between the Two Areas
V	±0.0062 0.0017–0.024	±2.40 0.92–9.29	*p* = 0.0002 ***	99%
Cr	±0.031 0.00–0.43	±2.16 0.93–8.37	*p* = 0.0002 ***	98%
Co	±0.012 0.0079–0.16	±0.33 0.13–1.28	*p* = 0.0003 ***	96%
Ni	±0.068 0.00–1.09	±1.38 0.87–6.32	*p* = 0.0004 ***	95%
As	±0.0018 0.00–0.0044	±0.19 0.082–0.80	*p* = 0.0002 ***	99%
Se	±0.13 0.038–0.17	±0.31 0.24–0.37	*p* = 0.0002 ***	58%
Cd	±0.00042 0.00–0.0013	±0.026 0.0094–0.10	*p* = 0.0001 ***	98%
Sn	±0.00 0.00–0.018	±0.20 0.13–5.36	*p* = 0.0012 **	100%
Pb	±0.059 0.018–0.28	±0.86 0.52–3.29	*p* = 0.0002 ***	93%

**Table 8:** Comparison between the metal concentrations in unwashed and washed feathers from P. D’Orleans. Only metals showing moderately significant (*p* < 0.05 *), significant (*p* < 0.01 **), or highly significant (*p* < 0.001 ***) results were reported.

Metal	Unwashed Feathers P.D’Orleans ±Median min–max (ppm)	Washed Feathers P. D’Orleans ±Median min–max (ppm)	Significance (*p* Value)	Percentage Difference between the Two Areas
Cr	±0.93 0.40–2.18	±0.26 0.00–0.86	*p* = 0.006 **	72%
Mn	±11.50 7.48–37.70	±3.46 0.23–6.82	*p* = 0.0002 ***	72%
Co	±0.12 0.048–0.34	±0.060 0.0050–0.14	*p* = 0.015 *	51%
Cu	±9.42 7.63–10.86	±5.42 0.23–6.27	*p* =0.0002 ***	42%
Zn	±109.65 68.79–142.87	±39.39 1.64–58.56	*p* = 0.0002 ***	64%
As	±0.16 0.048–0.55	±0.067 0.0060–0.20	*p* = 0.041 *	58%
Se	±0.46 0.27–0.66	±0.36 0.011–0.45	*p* = 0.019 *	20%
Cd	±0.017 0.0081–0.047	±0.0045 0.00–0.013	*p* = 0.0009 ***	73%
Sn	±1.28 0.30–2.68	±0.15 0.0070–0.41	*p* = 0.0003 ***	88%
Pb	±2.43 1.02–7.07	±0.75 0.061–1.59	*p* = 0.0009 ***	68%

**Table 9:** Comparison between the metal concentrations in unwashed and washed feathers from Monreale. Only metals showing significant (*p* < 0.01 **), or highly significant (*p* < 0.001 ***) results were reported.

Metal	Unwashed Feathers Monreale ±Median min–max (ppm)	Washed Feathers Monreale ±Median min–max (ppm)	Significance (*p* Value)	Percentage Difference between the Two Areas
Al	±1260.76 550.71–5668.88	±142.75 26.20–1799.51	*p* =0.0025 **	89%
V	±2.40 0.92–9.29	±0.23 0.043–3.11	*p* = 0.0025 **	90%
Cr	±2.16 0.93–8.37	±0.22 0.043–2.96	*p* = 0.0025 **	89%
Mn	±15.43 6.33–60.63	±0.23 0.094–2.89	*p* = 0.0002 ***	98%
Fe	±863.91 344.35–4217.01	±98.97 27.15–1476.18	*p* = 0.0041 **	88%
Co	±0.33 0.13–1.28	±0.022 0.0075–0.43	*p* = 0.0025 **	93%
Ni	±1.38 0.87–6.32	±0.10 0.023–1.39	*p* = 0.0007 ***	92%
Cu	±7.44 5.75–12.77	±0.68 0.18–1.70	*p* = 0.0002 ***	90%
Zn	±97.12 71.91–173.87	±4.51 0.96–9.58	*p* = 0.0002 ***	95%
As	±0.19 0.082–0.80	±0.016 0.0070–0.18	*p* = 0.0005 ***	91%
Se	±0.31 0.24–0.37	±0.062 0.020–0.12	*p* = 0.0002 ***	80%
Cd	±0.026 0.0094–0.10	±0.00043 0.00022–0.0033	*p* = 0.0002 ***	98%
Sn	±0.20 0.13–5.36	±0.036 0.0086–0.10	*p* = 0.0002 ***	82%
Sb	±0.026 0.011–0.082	±0.0041 0.00–0.54	*p* = 0.0018 **	84%
Pb	±0.86 0.52–3.29	±0.053 0.021–0.41	*p* = 0.0002 ***	93%

## Data Availability

Not applicable.

## References

[1] Budnik L.T., Baur X. (2009). The Assessment of Environmental and Occupational Exposure to Hazardous Substances by Biomonitoring. Dtsch. Ärztebl. Int..

[2] Amadi C.N., Frazzoli C., Orisakwe O.E. (2020). Sentinel Species for Biomonitoring and Biosurveillance of Environmental Heavy Metals in Nigeria. J. Environ. Sci. Health Part C.

[3] Cullen P. (1990). Biomonitoring and Environmental Management. Environ. Monit. Assess..

[4] Directive 2010/63/EU Of the European Parliament and of the Council of 22 September 2010 on the Protection of Animals Used for Scientific Purposes Text with EEA Relevance. OJ L 276, 20.10.2010, p. 33–79. https://eur-lex.europa.eu/legal-content/EN/TXT/?uri=celex%3A32010L0063.

[5] Fossi M.C. (1994). Nondestructive Biomarkers in Ecotoxicology. Environ. Health Perspect..

[6] Justino C., Duarte A., Rocha-Santos T. (2017). Recent Progress in Biosensors for Environmental Monitoring: A Review. Sensors.

[7] Zhou Q., Zhang J., Fu J., Shi J., Jiang G. (2008). Biomonitoring: An Appealing Tool for Assessment of Metal Pollution in the Aquatic Ecosystem. Anal. Chim. Acta.

[8] Thakur S., Dhyani S., Bramhanwade K., Pandey K.K., Bokade N., Janipella R., Pujari P. (2020). Non-Invasive Biomonitoring of Mercury in Birds near Thermal Power Plants: Lessons from Maharashtra, India. Environ. Monit. Assess..

[9] Wu X., Cobbina S.J., Mao G., Xu H., Zhang Z., Yang L. (2016). A Review of Toxicity and Mechanisms of Individual and Mixtures of Heavy Metals in the Environment. Environ. Sci. Pollut. Res..

[10] Hanfi M.Y., Mostafa M.Y.A., Zhukovsky M.V. (2020). Heavy Metal Contamination in Urban Surface Sediments: Sources, Distribution, Contamination Control, and Remediation. Environ. Monit. Assess..

[11] Kim R.-Y., Yoon J.-K., Kim T.-S., Yang J.E., Owens G., Kim K.-R. (2015). Bioavailability of Heavy Metals in Soils: Definitions and Practical Implementation—A Critical Review. Environ. Geochem. Health.

[12] Fu Z., Xi S. (2020). The Effects of Heavy Metals on Human Metabolism. Toxicol. Mech. Methods.

[13] Järup L. (2003). Hazards of Heavy Metal Contamination. Br. Med. Bull..

[14] Abdullah M., Fasola M., Muhammad A., Malik S.A., Bostan N., Bokhari H., Kamran M.A., Shafqat M.N., Alamdar A., Khan M. (2015). Avian Feathers as a Non-Destructive Bio-Monitoring Tool of Trace Metals Signatures: A Case Study from Severely Contaminated Areas. Chemosphere.

[15] Lemly A.D. (1995). A Protocol for Aquatic Hazard Assessment of Selenium. Ecotoxicol. Environ. Saf..

[16] Solgi E., Mirzaei-Rajeouni E., Zamani A. (2020). Feathers of Three Waterfowl Bird Species from Northern Iran for Heavy Metals Biomonitoring. Bull. Environ. Contam. Toxicol..

[17] Furness R.W., Furness R.W., Greenwood J.J.D. (1993). Birds as Monitors of Pollutants. Birds as Monitors of Environmental Change.

[18] Malik R.N., Zeb N. (2009). Assessment of Environmental Contamination Using Feathers of *Bubulcus Ibis* L., as a Biomonitor of Heavy Metal Pollution, Pakistan. Ecotoxicology.

[19] Scheuhammer A.M. (1987). The Chronic Toxicity of Aluminium, Cadmium, Mercury, and Lead in Birds: A Review. Environ. Pollut..

[20] Binkowski Ł.J., Sawicka-Kapusta K., Szarek J., Strzyżewska E., Felsmann M. (2013). Histopathology of Liver and Kidneys of Wild Living Mallards Anas Platyrhynchos and Coots Fulica Atra with Considerable Concentrations of Lead and Cadmium. Sci. Total Environ..

[21] Braune B.M., Scheuhammer A.M. (2008). TRACE ELEMENT AND METALLOTHIONEIN CONCENTRATIONS IN SEABIRDS FROM THE CANADIAN ARCTIC. Environ. Toxicol. Chem..

[22] Di Giulio R.T., Scanlon P.F. (1984). Heavy Metals in Tissues of Waterfowl from the Chesapeake Bay, USA. Environ. Pollut. Ser. Ecol. Biol..

[23] Garcá-Fernández A.J., Sanchez-Garcia J.A., Gomez-Zapata M., Luna A. (1996). Distribution of Cadmium in Blood and Tissues of Wild Birds. Arch. Environ. Contam. Toxicol..

[24] Ansara-Ross T.M., Ross M.J., Wepener V. (2013). The Use of Feathers in Monitoring Bioaccumulation of Metals and Metalloids in the South African Endangered African Grass-Owl (Tyto Capensis). Ecotoxicology.

[25] Karimi M.-H.S., Hassanpour M., Pourkhabbaz A.-R., Błaszczyk M., Paluch J., Binkowski Ł.J. (2016). Trace Element Concentrations in Feathers of Five Anseriformes in the South of the Caspian Sea, Iran. Environ. Monit. Assess..

[26] Kertész V., Bakonyi G., Farkas B. (2006). Water Pollution by Cu and Pb Can Adversely Affect Mallard Embryonic Development. Ecotoxicol. Environ. Saf..

[27] Borghesi F., Migani F., Andreotti A., Baccetti N., Bianchi N., Birke M., Dinelli E. (2016). Metals and Trace Elements in Feathers: A Geochemical Approach to Avoid Misinterpretation of Analytical Responses. Sci. Total Environ..

[28] Goutner V., Furness R.W., Papakostas G. (2001). Mercury in Feathers of Squacco Heron (Ardeola Ralloides) Chicks in Relation to Age, Hatching Order, Growth, and Sampling Dates. Environ. Pollut..

[29] Frazzoli C., Bocca B., Mantovani A. (2015). The One Health Perspective in Trace Elements Biomonitoring. J. Toxicol. Environ. Health Part B.

[30] https://store.uni.com/p/CEN11013888/en-138052002-197145/CEN11013888_OEN.

[31] Craighead D., Bedrosian B. (2008). Blood Lead Levels of Common Ravens With Access to Big-Game Offal. J. Wildl. Manag..

[32] Commission Decision Of 12 August 2002 Implementing Council Directive 96/23/EC Concerning the Performance of Analytical Methods and the Interpretation of Results (Text with EEA Relevance) (2002/657/EC) (Notified under Document Number C(2002) 3044). https://eur-lex.europa.eu/legal-content/EN/ALL/?uri=CELEX%3A32002D0657.

[33] Commission Regulation (EU) No 836/2011 of 19 August 2011 Amending Regulation (EC) No 333/2007 Laying down the Methods of Sampling and Analysis for the Official Control of the Levels of Lead, Cadmium, Mercury, Inorganic Tin, 3-MCPD and Benzo(a)Pyrene in Foodstuffs Text with EEA Relevance. OJ L 215, 20.8.2011, p. 9–16. https://eur-lex.europa.eu/LexUriServ/LexUriServ.do?uri=OJ:L:2011:215:0009:0016:EN:PDF.

[34] Commission Regulation (EC) No 333/2007 of 28 March 2007 Laying down the Methods of Sampling and Analysis for the Official Control of the Levels of Lead, Cadmium, Mercury, Inorganic Tin, 3-MCPD and Benzo(a)Pyrene in Foodstuffs (Text with EEA Relevance). OJ L 88, 29.3.2007, p. 29–38. https://eur-lex.europa.eu/legal-content/EN/ALL/?uri=celex%3A32007R0333.

[35] https://eur-lex.europa.eu/legal-content/IT/TXT/PDF/?uri=CELEX:32014R0488&from=DE.

[36] https://eur-lex.europa.eu/legal-content/it/ALL/?uri=CELEX%3A32002D0657.

[37] https://store.uni.com/uni-cei-en-iso-iec-17025-2005.

[38] https://eur-lex.europa.eu/legal-content/IT/TXT/PDF/?uri=CELEX:32007R0333&from=LV.

[39] https://eur-lex.europa.eu/LexUriServ/LexUriServ.do?uri=OJ:L:2011:215:0009:0016:IT:PDF.

[40] https://store.uni.com/uni-en-15763-2010.

[41] Adout A., Hawlena D., Maman R., Paz-Tal O., Karpas Z. (2007). Determination of Trace Elements in Pigeon and Raven Feathers by ICPMS. Int. J. Mass Spectrom..

[42] Berglund Å.M.M. (2018). Evaluating Blood and Excrement as Bioindicators for Metal Accumulation in Birds. Environ. Pollut..

[43] Carneiro M., Colaço B., Brandão R., Ferreira C., Santos N., Soeiro V., Colaço A., Pires M.J., Oliveira P.A., Lavín S. (2014). Biomonitoring of Heavy Metals (Cd, Hg, and Pb) and Metalloid (As) with the Portuguese Common Buzzard (Buteo Buteo). Environ. Monit. Assess..

[44] Ek K.H., Morrison G.M., Lindberg P., Rauch S. (2004). Comparative Tissue Distribution of Metals in Birds in Sweden Using ICP-MS and Laser Ablation ICP-MS. Arch. Environ. Contam. Toxicol..

[45] Grúz A., Mackle O., Bartha A., Szabó R., Déri J., Budai P., Lehel J. (2019). Biomonitoring of Toxic Metals in Feathers of Predatory Birds from Eastern Regions of Hungary. Environ. Sci. Pollut. Res..

[46] Hanć A., Zduniak P., Erciyas-Yavuz K., Sajnóg A., Barałkiewicz D. (2017). Laser Ablation-ICP-MS in Search of Element Pattern in Feathers. Microchem. J..

[47] Mikoni N.A., Poppenga R., Ackerman J.T., Foley J., Hazlehurst J., Purdin G., Aston L., Hargrave S., Jelks K., Tell L.A. (2017). Trace Element Contamination in Feather and Tissue Samples from Anna’s Hummingbirds. Ecol. Indic..

[48] Willie S., Brophy C., Clancy V., Lam J., Sturgeon R., Yang L. (2012). DORM-4: Matériau de Référence Certifié de Protéines de Poissons Pour l’analyse Des Métaux à l’état de Traces. DORM-4: Fish Protein Certified Reference Material for Trace Metals.

[49] Boncompagni E., Muhammad A., Jabeen R., Orvini E., Gandini C., Sanpera C., Ruiz X., Fasola M. (2003). Egrets as Monitors of Trace-Metal Contamination in Wetlands of Pakistan. Arch. Environ. Contam. Toxicol..

[50] Burger J. (1993). Metals in Feathers of Brown Noddy (Anous Stolidus): Evidence for Bioaccumulation or Exposure Levels?. Environ. Monit. Assess..

[51] Ritchie B.W., Harrison G.J., Harrison L.R. (1994). Avian Medicine: Principles and Application.

[52] Church H.J., Day J.P., Braithwaite R.A., Brown S.S. (1993). Binding of Lead to a Metallothionein-like Protein in Human Erythrocytes. J. Inorg. Biochem..

[53] Edwards W.R., Smith K.E. (1984). Exploratory Experiments on the Stability of Mineral Profiles of Feathers. J. Wildl. Manag..

[54] Buggiani S.S., Rindi S. (1980). Lead Toxicosis and Salt Glands in Domestic Ducks. Bull. Environ. Contam. Toxicol..

